# Tinzaparin vs. Nadroparin Safety and Efficacy in Neurosurgery

**DOI:** 10.3390/neurolint13020021

**Published:** 2021-05-13

**Authors:** Florian Wilhelmy, Annika Hantsche, Michael Gaier, Johannes Kasper, Michael Karl Fehrenbach, Rene Oesemann, Jürgen Meixensberger, Dirk Lindner

**Affiliations:** 1 Department of Neurosurgery, University Hospital Leipzig, Liebigstrasse 20, 04103 Leipzig, Germany; Annika.hantsche@medizin.uni-leipzig.de (A.H.); mg50lino@studserv.uni-leipzig.de (M.G.); johannes.kasper@medizin.uni-leipzig.de (J.K.); michael.fehrenbach@medizin.uni-leipzig.de (M.K.F.); juergen.meixensberger@medizin.uni-leipzig.de (J.M.); dirk.lindner@medizin.uni-leipzig.de (D.L.); 2 Department of Anesthesiology and Intensive Care, University Hospital Leipzig, Liebigstrasse 20, 04103 Leipzig, Germany; rene.oesemann@medizin.uni-leipizg.de

**Keywords:** tinzaparin, African swine fever, heparin shortage, nadroparin, neurosurgery, perioperative anticoagulation

## Abstract

Background: An outbreak of African swine fever (ASF) in China in 2020 has led to an unprecedented shortage of nadroparin. Most patients, especially those kept in hospital for surgery, are currently treated with prophylactic anticoagulation (AC). In search of alternatives for nadroparin (fraxiparine), we found no sufficient data on alternatives for neurosurgical patients, such as tinzaparin of European origin. We compared nadroparin and tinzaparin concerning adverse events (bleeding versus thromboembolic events) in neurosurgical patients. Methods: Between 2012 and 2018, 517 neurosurgical patients with benign and malignant brain tumors as well as 297 patients with subarachnoid hemorrhage (SAH) were treated in the Department of Neurosurgery, University Hospital Leipzig, receiving prophylactic anticoagulation within 48 h. In 2015, prophylactic anticoagulation was switched from nadroparin to tinzaparin throughout the university hospital. In a retrospective manner, the frequency and occurrence of adverse events (rebleeding and thromboembolic events) in connection with the substance used were analyzed. Statistical analysis was performed using Fisher’s exact test and the chi-squared test. Results: Rebleeding rates were similar in both nadroparin and tinzaparin cohorts in patients being treated for meningioma, glioma, and SAH combined (8.8% vs. 10.3%). Accordingly, the rates of overall thromboembolic events were not significantly different (5.5% vs. 4.3%). The severity of rebleeding did not vary. There was no significant difference among subgroups when compared for deep vein thrombosis (DVT) or pulmonary embolism (PE). Conclusion: In this retrospective study, tinzaparin seems to be a safe alternative to nadroparin for AC in patients undergoing brain tumor surgery or suffering from SAH.

## 1. Introduction

**Figure 1:**
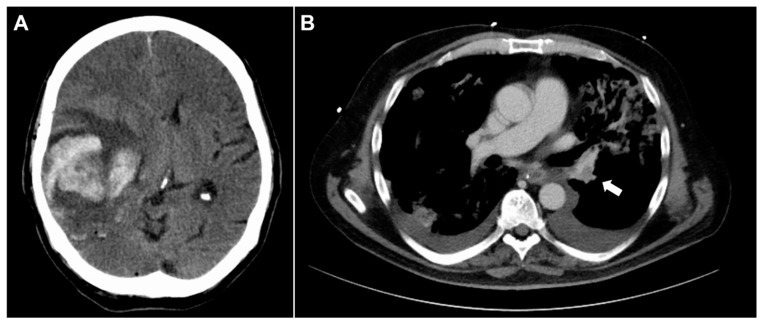
(**A**) Computed tomography (CT) scan of a postoperative rebleeding after glioma resection. (**B**) Pulmonary embolism is a complication commonly seen in neurosurgical patients.

All hospitalized patients are currently treated with prophylactic anticoagulation (AC) according to guidelines [1,2,3]. (Neuro-)surgical patients are at elevated risk of both thromboembolic events and intracranial rebleeding [4,5,6] (Figure 1). Data on low molecular weight heparin (LMWH) safety is therefore essential for safe prophylactic treatment. Due to an outbreak of African swine fever (ASF) in China [7], heparin doses are at risk of running short. Many hospitals use LMWH of Chinese production. Should there be an actual shortage, alternatives are required to maintain supply. Quite recently, the first case of ASF in wildlife was discovered in Germany, indicating a westward movement of the disease [8,9].

In our facility, anticoagulation was formerly administered using nadroparin. For various reasons, in 2015, the preferred medication was switched to tinzaparin, which is made in Europe. Tinzaparin supplies therefore need to be relatively independent of local ASF outbreaks [6].

Coincidentally, we retrospectively analyzed patient data on prophylactic anticoagulation from 2012 to 2018 [10], dividing the AC-treated cohorts almost equally between nadroparin and tinzaparin. We considered a heterogenous collective of patients with acute, malignant, and benign disease as representative. In this study, we present data directly comparing tinzaparin and nadroparin in prophylactic doses regarding adverse events such as rebleeding as well as postoperative hemorrhage and thromboembolic events (TEs).

## 2. Methods

The study was approved by the ethics committee of the Medical Faculty, University of Leipzig (No. 053/19-ek).

### 2.1. Patient Selection

This retrospective study included 517 patients undergoing brain tumor surgery (278 meningioma, 239 malignant brain tumors) and 297 suffering from subarachnoid hemorrhage (SAH) treated at the Department of Neurosurgery, University Hospital Leipzig, between 2012 and 2018. Selection was carried out via ICD-10 (International Statistical Classification of Diseases and Related Health Problems) registration in the hospital database. Inclusion criteria were age above 18 years and conclusive documentation as well as prophylactic anticoagulation within 48 h after operation or hospital admission.

### 2.2. Diagnosis of Adverse Events

Cranial computer tomography (CT) or magnetic resonance imaging (MRI) scans were performed within 6 h after the initial event and within 24 h after surgery or intervention. Patients showing new neurological deficits or unsatisfactory wake-up reaction after intervention underwent CT scans.

Diagnostic methods (e.g., duplex sonography of the veins, CT scan of the chest or abdomen) for systemic thromboembolic events were only performed if symptoms occurred.

### 2.3. Anticoagulation Protocol for SAH Patients at Our Clinic

Decisions on the type and dosage of anticoagulation were based on current guidelines [1,2,3] and interdisciplinary bedside rounds. LMWH doses were adjusted for body weight. The standard dosage was 4500 IE (tinzaparin) or 2850 IE (nadroparin). Patients routinely received heparin within 48 h. Reasons for no or delayed prophylactic AC were foudroyant rebleeding, death, or treatment with flow-diverting devices or a stent necessitating different AC. Moreover, patients suffering from acute, life-threatening embolisms, such as pulmonary embolism, were therapeutically anticoagulated immediately.

### 2.4. Assessed Data

Assessed data included biographic data, primary endpoints, and comorbidities and complications, as detailed below.

Biographic: gender, age, body mass index (BMI), smoking, pre-existing hypertension, pre-existing therapeutic anticoagulation

Primary endpoints: intracranial rebleeding, thromboembolic event (cerebral and systemic)

Comorbidities and complications: dialysis, steroid medication, acute disorder of lungs or heart, coagulation disorder

### 2.5. Statistical Analysis

To describe the cohort, nominal parameters are displayed as percentages.

The population was dichotomously classified depending on the occurrence or nonoccurrence of adverse events (intracranial rebleeding, systemic thromboembolic event, and cerebral thromboembolic event).

Dichotomous parameters were analyzed using the chi-squared test or Fisher’s exact test. Continuous data was analyzed with *t*-test, normality test was performed. In this study, *p*-values below 0.05 were considered statistically significant.

All analyses were computed using GraphPad QuickCalcs (www.graphpad.com, accessed on 09 November 2020) (GraphPad Software, LLC, San Diego, CA, USA).

## 3. Results

Eight hundred and fourteen patients received prophylactic heparinization within 48 h. Patients’ cohorts showed typical distribution of demographic data and pre-existing medical conditions compared to typical cohorts of the specific disease (Table 1). Cohorts showed no significant differences in baseline data. Patients with pre-existing anticoagulation (*n* = 104, 12.8%) were not excluded from the cohorts. We performed subgroup analysis for patients with pre-existing anticoagulation for meningioma, SAH, and glioma individually. For meningioma and glioma surgery, therapeutic AC was paused for surgery. Logically, we found no effect on adverse events. In SAH, however, pre-existing anticoagulation prior to the initial event increased the risk of intracranial rebleeding (*p* = 0.009, OR 2.417, 95% CI of OR 1.278–4.570). This effect turned out to be insignificant when groups were combined. In all SAH patients combined, there was no difference between tinzaparin and nadroparin groups (*p* = 0.7101).

Eight hundred and fourteen patients were treated prophylactically with either nadroparin (*n* = 398) or tinzaparin (*n* = 416). Regarding hemorrhagic complications, we divided patients between those who did or did not undergo surgical treatment of rebleeding to gauge bleeding severity. The reasons for delayed or non-prophylactic treatment were severe rebleeding, acute pulmonary embolism, or securing aneurysm with a stent or flow-diverting devices, making therapeutic AC necessary. After combining the heterogenous groups of patients, differences between nadroparin and tinzaparin were not significant. No differences were observed regarding bleeding complications nor thromboembolic events (Table 2).

## 4. Discussion

Tinzaparin has been proven to be effective and safe in various patients [11,12,13]. Data on neurosurgical patients are especially scarce and do not compare specific substances nor different diseases [14,15].

The study is limited due to its retrospective character. Although the number of examined patients is relatively high (*n* = 814), the study is not sufficiently powered to securely exclude differences between the substances investigated. Patient groups are heterogenous regarding underlying disease. Despite differences in the underlying pathomechanism of rebleeding and thromboembolism [16,17], we assume that the combined cohort is applicable to heterogenous neurosurgical patient collectives. When dividing into different subgroups regarding underlying disease, we did not find specific differences.

As AC is obligatory under current guidelines, we have no control group that is not treated with AC. For obvious reasons, we cannot draw any conclusions regarding the general safety or efficacy of prophylactic AC.

## 5. Conclusions

We suppose the application of tinzaparin in neurosurgical patients to be as safe as nadroparin. According to our data, tinzaparin is a suitable alternative to other anticoagulative substances and so could be used to counter supply shortages.

## Figures and Tables

**Table 1:** Baseline data. Groups are comparable regarding demographic and medical data.

Patients’ Baseline Data (*n* = 814)			
Demographic	Nadroparin (*n* = 398)	Tinzaparin (*n* = 416)	*p*-Value (*t*-Test/Fisher’s Exact)
Gender	167 M (42.0%)231 F (58.0%)	182 M (43.8%)234 F (56.2%)	0.6205
Age (average in years)	59 ± 15.0	60 ± 14.6	0.3354
BMI (average)	26 ± 4.5	26 ±4.6	1.000
Underlying disease			
Malign brain tumor	106 (26.6%)	133 (32.0%))	0.0906
Meningioma	149 (37.4%)	129 (31.0%)	0.0550
SAH	143 (35.9%)	154 (37.0%)	0.7710
Medical history			
Pre-existing anticoagulation	49 (12.3%)	55 (13.2%)	0.7530
Smoker	71 (17.8%)	80 (19.2%)	0.6523
Steroid medication	196 (49.2%)	190 (45.7%)	0.3259
Coagulation disorder	35 (8.8%)	34 (8.2%)	0.8018

SAH: subarachnoid hemorrhage. M: male. F: female. BMI: body mass index.

**Table 2:** Adverse events of the entire cohort treated with prophylactic nadroparin or tinzaparin. Eight hundred and fourteen patients received prophylactic anticoagulation.

Adverse Events Combined (*n* = 814)				
Adverse Event	Nadroparin (*n* = 398)	Tinzaparin (*n* = 416)	*p*-Value	
			Chi-Squared	Fisher’s Exact
Rebleeding conservative (overall)	35 (8.8%)	43 (10.3%)	0.4996	0.4757
Rebleeding operative	13 (3.3%)	11 (2.6%)	0.7551	0.6818
Thromboembolic event (overall)	22 (5.5%)	18 (4.3%)	0.5287	0.517
Deep vein thrombosis	8 (2.0%)	4 (0.96%)	0.3513	0.2561
Pulmonary embolism	9 (2.3%)	9 (2.2%)	0.9417	1
Systemic thromboembolism	5 (1.3%)	5 (1.20%)	0.9568	1

## Data Availability

Raw data were generated at University Hospital Leipzig. Derived data supporting the findings of this study are available from the corresponding author FW on request.
